# Corrigendum to: Stattic sensitizes osteosarcoma cells to epidermal growth factor receptor inhibitors via blocking the interleukin 6-induced STAT3 pathway

**DOI:** 10.3724/abbs.2025079

**Published:** 2025-06-25

**Authors:** Shenglin Wang, Yunqing Wang, Zhen Huang, Hongxiang Wei, Xinwen Wang, Rongkai Shen, Wenbin Lan, Guangxian Zhong, Jianhua Lin

*Acta Biochim Biophys Sin* ( *Shanghai*) *2021*, *53*( *12*): *1670*– *1680*

http://doi: 10.1093/abbs/gmab146

In the original version of this manuscript, an error was found in Figure 2 and Figure 6 respectively. The correct figures are shown as follows. The authors apologize for the error.

**Figure:**
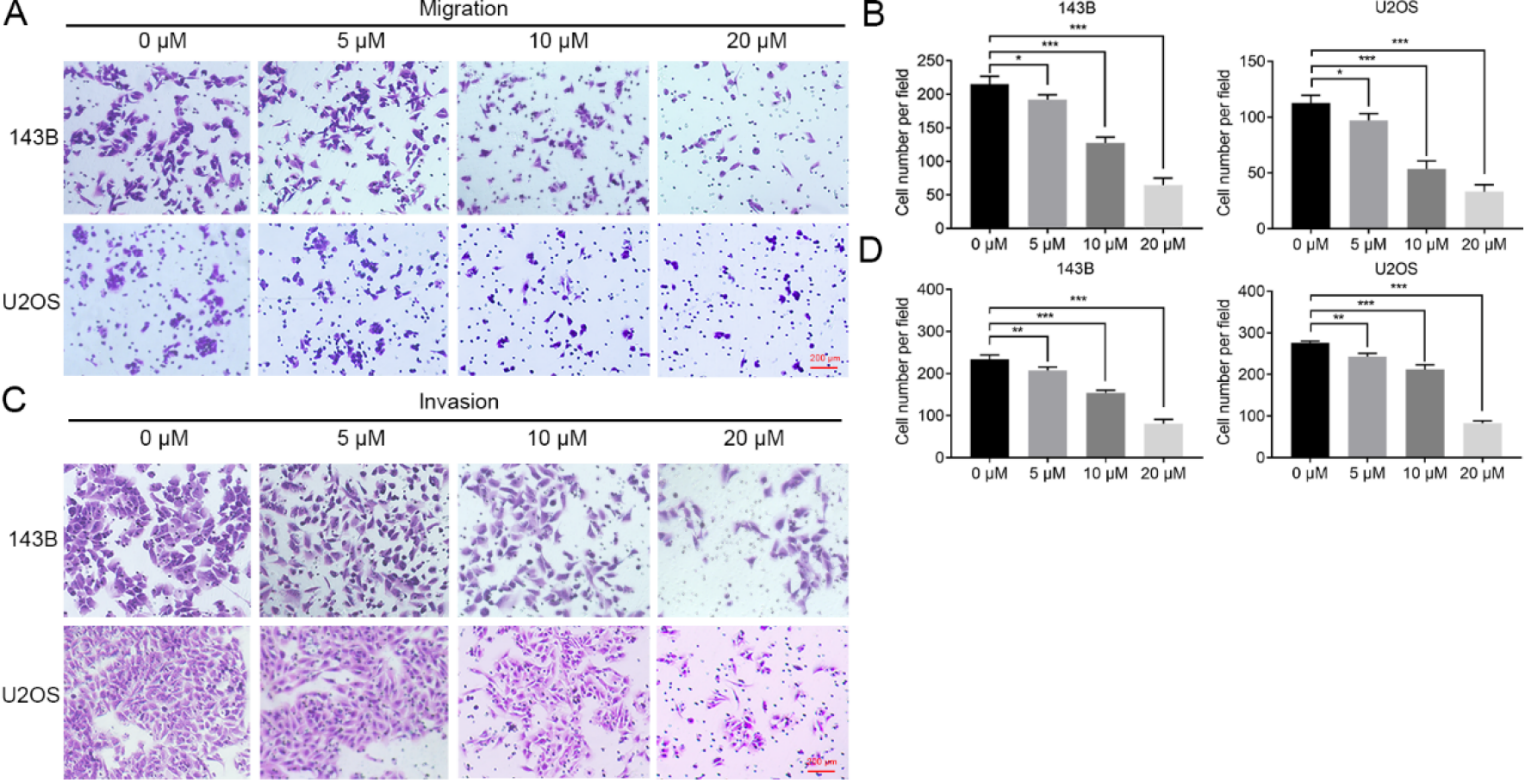
Figure 2 Gefitinib decreased the migration and invasion of OS cells The number of migrated (A,B) and invasive (C,D) 143B and U2OS cells determined by the transwell assay after gefitinib or DMSO treatment. Magnification: 200×. *P < 0.05, **P < 0.01, ***P < 0.001.

**Figure:**
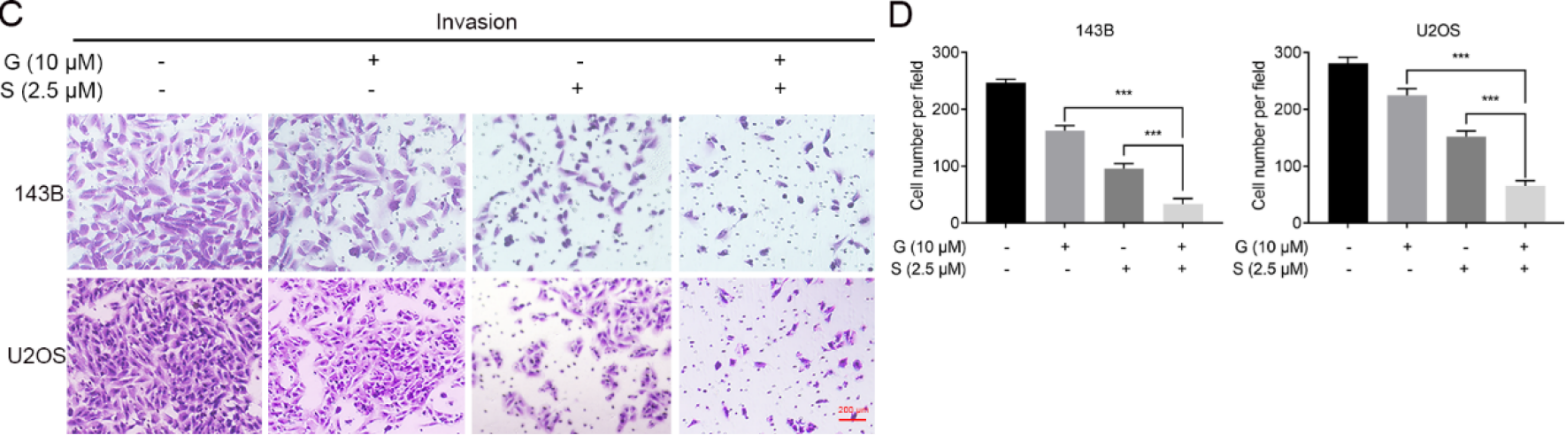
Figure 6 Stattic promoted the inhibitory effect of gefitinib on the metastasis of OS cells (C,D) The number of invasive 143B and U2OS cells determined by transwell assay after gefitinib, stattic, or the dual treatment. G: gefitinib; S: stattic. Magnification: 200×. ***P < 0.001.

